# Subconjunctival Injection of Viscoelastic Material for Leaking Sclerotomy in Transconjunctival Sutureless Vitrectomy

**DOI:** 10.1155/2016/9659675

**Published:** 2016-04-06

**Authors:** Chung Hyun Lee, Soo Geun Joe, Sung Jae Yang

**Affiliations:** Department of Ophthalmology, Gangneung Asan Hospital, College of Medicine, University of Ulsan, Gangneung 25440, Republic of Korea

## Abstract

*Aim.* To evaluate the effectiveness of subconjunctivally injected viscoelastic material (VEM) for the self-sealing of leaking sclerotomy in transconjunctival sutureless vitrectomy (TSV).* Methods*. This was a prospective interventional series. Subconjunctival injection of VEM was performed in eyes showing leaking sclerotomy at the end of TSV in selected cases. This procedure was performed in 24 consecutive eyes from 24 patients scheduled for 23- or 25-gauge TSV with phacoemulsification for various vitreoretinal diseases combined with cataracts.* Results.* Among the 24 eyes, 13 cases were scheduled for 23-gauge TSV, while 11 cases were scheduled for 25-gauge TSV. The average number of injection sites per eye was 1.7 ± 0.9 in the 23-gauge cases and 1.5 ± 0.7 in the 25-gauge cases. Leakage was most commonly observed at the vitrector site of the sclerotomy, while little leakage was observed at the illuminator site. There were no cases of postoperative hypotony.* Conclusion.* Subconjunctival injection of VEM was simple and effective for the self-sealing of leaking sclerotomy after TSV in selected cases.

## 1. Introduction

After their introduction by Machemer et al. [1] in 1971, vitrectomy procedures have been markedly improved by the development of more advanced machines and equipment. Accordingly, small incision sutureless vitrectomy is becoming increasingly popular for the surgical management of various vitreoretinal disorders [2, 3]. The concept of transconjunctival sutureless vitrectomy (TSV) suggests that this method may have some advantages over traditional vitrectomy, including reduced surgical trauma, less postoperative discomfort, faster visual recovery, shorter operation time, and reduced postoperative astigmatism [2, 4–6]. However, even with recent advancements in incision techniques, such as two-step sclera tunnel incision, oblique incision, and slit-shaped sclera tunnel incision for avoiding wound leakage [3, 7–11], complete self-sealing of all sclerotomy sites is still challenging.

**Figure 1:**
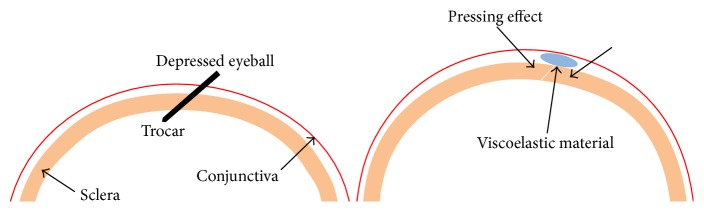
Schematic showing the mechanism of subconjunctival viscoelastic material injection. Flattening of the globe and conjunctival slippage occurred, and there was slight displacement of entry site between the conjunctiva and sclera. Subconjunctival injection of viscoelastic material had a transient tamponade effect on the sclerotomy site following pressing of the sclera beneath the conjunctiva.

In cases of leaking sclerotomy, most surgeons insert transscleral and transconjunctival absorbable sutures. Many studies have described attempts to manage leaking sclerotomy, such as air tamponade [12, 13], the releasable suture technique [14], transconjunctival plain cut tape [15], fibrin glue application [16], and diathermy of leaking sclerotomy [17–19]. In our institution, when we perform a trocar incision using the two-step sclera tunnel or oblique incision technique, flattening of the globe and conjunctival slippage occur, with slight displacement of the entry site between the conjunctiva and sclera (Figure 1). In a study by Lee and Song, no wound leakage was observed, even at 4 h after removal of releasable sutures [14]. Therefore, we hypothesized that subconjunctivally injected viscoelastic material (VEM) would have a transient tamponade effect on the sclerotomy site by pressing the sclera beneath the conjunctiva, ensuring wound closure without sutures (Figure 1).

The aim of this study was to evaluate the effectiveness of subconjunctivally injected VEM for the prevention of incompetent wound closure in TSV.

## 2. Methods

### 2.1. Patients and Study Design

This study was performed after approval by the Institutional Review Board of Gangneung Asan Hospital. We carried out subconjunctival injection of VEM into eyes showing leaking sclerotomy at the end of vitrectomy in selected cases. However, for cases of profuse leaking sclerotomy, absorbable sutures were placed. This procedure was performed in 24 consecutive eyes from 24 patients scheduled for 23- or 25-gauge transconjunctival vitrectomy with phacoemulsification for various vitreoretinal diseases combined with cataracts. All the patients included in this study underwent combined phacoemulsification and posterior intraocular lens insertion, and the remaining VEM during the cataract operation was used for the subconjunctival injection. We evaluated the location and number of leaking sites per eye.

### 2.2. Surgical Technique

All operations were performed at one hospital by the same surgeon (S. J. Yang) with an Accurus instrument (Alcon Laboratories, Inc., Fort Worth, TX, USA) using the Edge Plus trocar system (Alcon Laboratories, Inc.). A cohesive VEM (Healon GV, Abbott Medical Optics, Inc.) was used for all cases. For self-sealing of sclerotomy, incisions with trocars were created in a beveled (15°–20°) approach. The conjunctiva was displaced, and the sclera was penetrated 3.5 mm posterior to the limbus. A complete vitrectomy was carried out, including vitreous base shaving using a noncontact wide-field image system (BIOM, Oculus, Munich, Germany). The cannulas were removed by slowly pulling each cannula out along the entry path over the illuminator or vitrector. The illuminator or vitrector was then slowly removed, and each sclerotomy site was gently pressed with a cotton swab or blunt-angled forceps. When leakage was detected, subconjunctival injection of VEM was performed, and the wound was gently pressed and massaged. In all cases of subconjunctival VEM injection, there was no immediate leakage observed at the end of surgery. The eyes were dressed with ocular patches to provide gentle pressure. In cases of profuse leaking sclerotomy, absorbable sutures were placed; these cases were excluded from our study.

Patients were examined 1 day after the operation with an anterior slit lamp to evaluate the possible presence of leakage. Intraocular pressure was measured by noncontact tonometry.

Patients were examined one week and one month after the operation then.

## 3. Results

Of the 24 patients enrolled in this study, 15 were men. The mean age of the patients was 56.3 ± 11.8 years (Table 1). Among the 24 eyes, 13 were scheduled for 23-gauge vitrectomy, while 11 were scheduled for 25-gauge vitrectomy. Vitreous hemorrhage was the most common surgical indication for vitrectomy, followed by macular disorders. Silicone oil tamponade was performed in six eyes, and gas tamponade was performed in two eyes.

Among 24 eyes, 14 eyes were subconjunctivally injected with VEM at one sclerotomy site. The average number of injection sites per eye was 1.7 ± 0.9 in the 23-gauge group and 1.5 ± 0.7 in the 25-gauge group (Table 2).

We defined the leaking sclerotomy sites as vitrector (superotemporal in the right eye or superonasal in the left eye), illuminator (superotemporal in the left eye or superonasal in the right eye), and infusion (inferotemporal). Leakage was most common at the vitrector site, often requiring VEM injection. The least leakage was observed at the illuminator site (Table 2).

**Figure 2:**
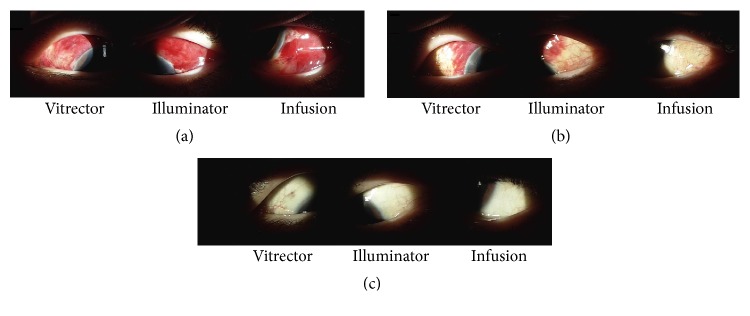
Postoperative anterior segment photos from operation in a 52-year-old woman. (a) Mild chemosis and subconjunctival hemorrhage were observed at all three areas, and the intraocular pressure was 23 mmHg on postoperative day 1. (b) Subconjunctival hemorrhage decreased, and the intraocular pressure was 17 mmHg at 1 week after operation. (c) Anterior slit lamp photos showed clear conjunctiva at 1 month after operation, and the intraocular pressure was 18 mmHg.

**Figure 3:**
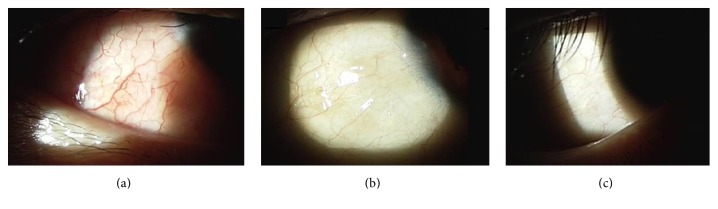
Representative anterior segment photography of a 42-year-old man who underwent 23-gauge vitrectomy with air tamponade due to diabetic vitreous hemorrhage. (a) Mild chemosis was observed at the inferotemporal quadrant with mild conjunctival injection in anterior segment photographs on postoperative day 1. (b) Conjunctival chemosis and injection disappeared at 1 week after operation. (c) Clear conjunctiva was observed at 1 month after operation.

Two representative cases of subconjunctival VEM injection are shown in Figures 2 and 3. As shown in Figure 2, a 52-year-old woman with diabetic vitreous hemorrhage in the left eye underwent 23-gauge vitrectomy. At the end of vitrectomy, sclerotomy leakage was observed in all three areas. Subconjunctival injection of VEM was then performed at all three areas. In the second case (Figure 3), a 42-year-old man with diabetic vitreous hemorrhage in his right eye underwent 23-gauge vitrectomy and air tamponade. VEM material was injected at the infusion site. There were no cases of postoperative hypotony (Table 3).

## 4. Discussion

The aim of this study was to evaluate the effectiveness of a new approach for the prevention of leaking sclerotomy in TSV. Importantly, we found that subconjunctival injection of VEM was effective for the self-sealing of leaking sclerotomy after TSV in selected cases. Thus, this method may have important applications.

Wound leakage or hypotony, even if transient, may lead to serious complications, such as endophthalmitis, suprachoroidal hemorrhage, choroidal detachment, and hypotony maculopathy [13, 20–22]. In particular cases, such as cases of myopia, reoperation, vitreous dissection, or multiple exchanges of surgical instruments, postoperative wound leakage is more frequent [4, 22, 23]. The incidence of leaking sclerotomy varies with the use of different surgical instruments, different surgical techniques, and different tamponading agents.

Patients included in this study underwent combined phacoemulsification and vitrectomy; the remaining VEM was used during phacoemulsification. All previously described methods have used extra materials or devices and additional tissue manipulation, with the exception of the air or gas tamponade method. Moreover, while bipolar diathermy of leaking sclerotomy is a quick and effective method, it has been shown to cause conjunctival scarring [18]. Additionally, fibrin glue-assisted conjunctival closure has also been shown to be effective and does not require tissue manipulation; however, this method is expensive and is associated with antigen reactions. Thus, because subconjunctival VEM injection did not require additional materials or devices, it provided major advantages over these other methods. The mechanism of wound closure in this method may be the tamponade effect of the subconjunctival placement of the VEM by depression and sealing of the outer surface of the sclerotomy (Figure 1) and blocking of the sclerotomy sites.

This method has several limitations. First, this technique cannot be used in all cases of vitrectomy; we would recommend that this method not be used for patients whose eyes have thin sclera and thin conjunctiva or profuse leaking sclerotomy or for patients undergoing reoperation. However, our results showed that this technique could be simple and effective for self-sealing of leaking sclerotomy in selected cases.

## 5. Conclusion

Subconjunctival injection of VEM can be simple and effective method for the self-sealing of leaking sclerotomy after TSV in selected cases.

## Supplementary Material

Video clip demonstrating the procedure of subconjuntival viscoelastic material injection in cases of leaking sclerotomy. After removal of surgical instrument, subtle leakage is noticed. Subconjunctivally injected visoelastic material prevents further sclerotomy leakage after small gauge vitrectomy surgery.

## Figures and Tables

**Table 1:** Patient demographics.

Age, mean ± SD, years (*n* = 24)	56.3 ± 11.8

Gender, *n* (%)	Male 15 (62.5%)
	Female 9 (37.5%)

Surgical indication, *n* (%)	
Vitreous hemorrhage	11 (45.8%)
Macular surgery	7 (29.2%)
Retinal detachment	3 (12.5%)
Tractional retinal detachment	3 (12.5%)

Tamponade material, *n* (%)	
Silicone oil	6 (25%)
Gas	2 (8%)
Air	1 (4%)
Balanced salt solution	15 (63%)

**Table 2:** The number and site of leaking sclerotomies.

	Vitrector	Illuminator	Infusion	Average/eye
25-gauge	9/11 eyes	2/11 eyes	5/11 eyes	1.5 ± 0.7
23-gauge	9/13 eyes	5/13 eyes	8/13 eyes	1.7 ± 0.9
Total	18/24 eyes	7/24 eyes	13/24 eyes	1.6 ± 0.8

**Table 3:** Postoperative changes in intraocular pressure.

	1 day	1 week	1 month
25-gauge	16.0 ± 3.0	15.4 ± 4.8	15.7 ± 2.6
23-gauge	17.8 ± 4.5	15.9 ± 7.3	14.7 ± 2.8
Total	16.9 ± 3.9	15.7 ± 6.1	15.2 ± 2.7
